# Nuclear Enlargement as a Histological Hallmark of Skeletal Muscle Aging, Revealed by Deep Learning‐Driven Analysis and Validated in Inflammatory Myopathies

**DOI:** 10.1111/acel.70577

**Published:** 2026-06-15

**Authors:** Tam Dao, Thanh T. Nguyen, Gia Minh Hoang, Junhyeon Park, Yunju Jo, Thach Hoang Ngoc, Diep Hong Pho, Dien Tran Minh, Emma Anh Ton, Sunjae Lee, Hyun Jin Kim, Vu Chi Dung, Jae Gwan Kim, Dongryeol Ryu

**Affiliations:** ^1^ Department of Biomedical Science and Engineering Gwangju Institute of Science and Technology (GIST) Gwangju Republic of Korea; ^2^ Department of Physiology Sungkyunkwan University School of Medicine Suwon Republic of Korea; ^3^ Center of Endocrinology, Metabolism, Genetic/Genomics and Molecular Therapy Vietnam National Children's Hospital Hanoi Vietnam; ^4^ Department of Physiology and Biomedical Engineering Mayo Clinic Scottsdale Arizona USA; ^5^ Department of Microbiology Wonkwang University School of Medicine Iksan Republic of Korea; ^6^ Department of Pathology Vietnam National Children's Hospital Ha Noi Vietnam; ^7^ Department of Computer Science Harvard John A. Paulson School of Engineering and Applied Sciences Boston Massachusetts USA; ^8^ Graduate School of Engineering Biology Korea Advanced Institute of Science & Technology (KAIST) Daejeon Republic of Korea; ^9^ Department of Pediatrics University of Medicine and Pharmacy—Vietnam National University Ha Noi Vietnam

**Keywords:** cellular senescence, deep learning, nuclear enlargement, skeletal muscle aging, transcriptomics

## Abstract

Aging reshapes the architecture of human skeletal muscle, yet objective tissue‐level markers that capture this process remain limited. We combined large‐scale histology with deep learning to identify reproducible features of muscle aging and to test their biological relevance. We analyzed 974 hematoxylin–eosin whole‐slide images from a population resource using a dual‐attention convolutional neural network and an independent Mask R‐CNN model to quantify nuclear size and density, verified by manual review. The classifier distinguished young from aged muscle with high accuracy (AUC 0.91; accuracy 86.2%), and attention maps consistently highlighted nuclear enlargement and spatial disorganization as salient features. Nuclear diameter increased with age (Spearman's *ρ* = 0.71, *p* < 0.0001) across automated and manual measurements. Transcriptomes matched to the same donors showed that samples with larger nuclei were enriched for pathways related to chromatin remodeling, proteostasis, cellular senescence, mitochondrial activity, and telomere regulation, whereas smaller nuclei aligned with anti‐inflammatory and DNA repair programs. External pediatric inflammatory myopathies exhibited nuclear enlargement comparable to aged muscle, suggesting inflammation‐related premature histologic aging. These findings identify nuclear enlargement as a robust, quantifiable feature that integrates structural and molecular signatures of muscle aging. The proposed deep learning–based nuclear morphometry provides a scalable framework for tissue‐level aging biomarkers and suggests a potential “muscle aging clock” applicable to both physiological aging and disease states.

## Graphical abstract

Deep learning–based analysis of human skeletal muscle histology identifies increased nuclear size and altered nuclear number as robust features of aging. These morphometric changes associate with coordinated transcriptional programs and support a scalable, tissue‐level biomarker framework for muscle aging, validated in inflammatory myopathy cohorts.

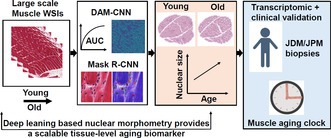

## 1 Introduction

Aging in skeletal muscle is characterized by progressive structural and functional decline, clinically manifesting as sarcopenia, reduced mobility, and increased frailty, significantly affecting quality of life among the elderly (Dao et al. 2020; Siparsky et al. 2014). At the tissue and cellular levels, aged skeletal muscle exhibits hallmark features, including muscle fiber atrophy, increased fibrosis, chronic inflammation (termed inflammaging), altered myonuclear organization, and compromised regenerative capacity (Gallay et al. 2020; Kedlian et al. 2024; Kunz and Lanza 2023; Lexell et al. 1988; Petrocelli et al. 2023; Siparsky et al. 2014). Recent investigations have increasingly highlighted the cell nucleus as a critical site of aging‐related remodeling. Studies in animal models and human cell lines have established that deterioration of nuclear morphology—including altered shape, architecture, and chromatin organization—is a hallmark of both senescence and physiological aging (Pathak et al. 2021; Scaffidi and Misteli 2006). In skeletal muscle specifically, aging is associated with significant molecular and structural alterations of the tissue nuclei (Cisterna and Malatesta 2024), affecting the cellular and subcellular components of the motor unit (Larsson et al. 2019). However, observations regarding nuclear size have been inconsistent across the literature; for instance, while some murine models demonstrate a decline in nuclear number and myonuclear domain size during age‐related atrophy (Brack et al. 2005), other studies report decreased myonuclear area, potentially due to species‐specific differences or variations in imaging methodologies, such as 2D histology versus 3D single‐fiber analysis (Cisterna and Malatesta 2024). Historically, histological studies on muscle aging have predominantly relied on manual evaluation to quantify changes in muscle fiber size, fiber‐type distribution, and extracellular matrix expansion (Granic et al. 2023; Jagtap et al. 2022; Kunz and Lanza 2023; Naruse et al. 2023; Siparsky et al. 2014). While these approaches have provided valuable insights, they are labor‐intensive, prone to observer bias, and inherently limited in their capacity to capture the complexity of tissue architecture. Consequently, a significant gap remains regarding the automated, high‐throughput, and unbiased characterization of aging‐related morphological features, particularly at the cellular and nuclear levels.

Recent advancements in imaging technologies and computational methodologies, especially artificial intelligence (AI)‐based techniques, present opportunities to systematically overcome the limitations of traditional histological analyses (Reis‐Filho and Kather 2023). Convolutional neural networks (CNNs), a class of deep learning algorithms, have demonstrated powerful capabilities in identifying subtle tissue alterations linked to disease progression and aging phenotypes across various organs (Chakradeo et al. 2024; Li et al. 2021). However, despite the increasing adoption of deep learning for muscle histology analysis, AI‐based approaches remain relatively limited in human skeletal muscle (Kabeya et al. 2022; Miazaki et al. 2015; Mill et al. 2025). In particular, deep learning methods have not yet been utilized to systematically characterize aging‐associated histological features or their relationships with underlying molecular changes, highlighting a significant knowledge gap in muscle aging research.

In this study, we aimed to establish an integrated deep learning‐based analytical pipeline for high‐throughput and unbiased analysis of skeletal muscle histology in aging. Leveraging large‐scale hematoxylin and eosin (H&E)‐stained muscle images from the Genotype‐Tissue Expression (GTEx) database, we developed and validated a dual attention module convolutional neural network (DAM‐CNN) designed to automatically detect aging‐associated morphological changes (Zhang 2022). We systematically quantified nuclear alterations, particularly nuclear enlargement—a known morphological hallmark of cellular senescence—using automated segmentation and manual validation methods to ensure accuracy and reproducibility (Hernandez‐Segura et al. 2018; Kuilman et al. 2010; Pathak et al. 2021). Additionally, to elucidate the biological significance of the identified morphological features, we integrated histological data with matched transcriptomic profiles, enabling a comprehensive exploration of molecular pathways linked to aging‐related nuclear changes.

We further validated the clinical and translational relevance of our findings by analyzing muscle biopsy samples from pediatric patients with inflammatory myopathies, which are characterized by muscle atrophy, degeneration, and chronic inflammation (Guglielmi et al. 2024; Jagtap et al. 2022; Liang et al. 2022). Premature nuclear enlargement observed in these patients revealed shared morphological features between disease‐associated muscle remodeling and physiological aging. This integrative approach links traditional histology with computational analysis, offering new insights into skeletal muscle aging. Our findings support nuclear enlargement as a robust histological hallmark and highlight the potential of nuclear morphometry as a tissue‐level biomarker—a histological “muscle aging clock”—for detecting premature aging phenotypes in both physiological and pathological contexts.

## 2 Materials and Methods

### 2.1 Preprocessing

#### 2.1.1 Histological Whole‐Slide Images (WSI)

Publicly available skeletal muscle histological WSIs were obtained from the GTEx portal on February 2024 (eGTEX Project 2017). The skeletal muscle samples were specifically collected from the gastrocnemius muscle, approximately 2 cm below the patella. Sample metadata included anonymized subject ID, sex, age bracket, hardy scale, pathology categories, and pathology notes. A total of 974 WSIs from donors aged from the 20s to 70s were included in the analysis.

#### 2.1.2 Hospital Histological Sample Collection

This study utilized archived skeletal muscle histological samples from 60 pediatric patients, collected between 2018 and 2024 at the Vietnam National Children's Hospital. Ethical approval for the use of these samples was obtained from the Institutional Review Board (IRB) of the Vietnam National Children's Hospital (IRB No. VN01037/IRB00011976/FWA00028418). All biopsies were performed at the Center for Endocrinology, Metabolism, Genomics, and Molecular Therapy, with tissue consistently sampled from clinically affected muscle regions. Biopsy specimens were subsequently processed and archived at the Department of Pathology following standard histological protocols. No additional biopsies were conducted specifically for this study.

The collected muscle biopsy samples were preserved in formalin and embedded in paraffin (FFPE) according to standard clinical protocols. Both previously stained and unstained histological sections were included in the study. Unstained sections were subjected to H&E staining using standard histological procedures. Briefly, 4 μm‐thick paraffin‐embedded tissue sections were deparaffinized in xylene, rehydrated through a graded ethanol series, and stained with Harris hematoxylin followed by Eosin Y. The stained sections were then dehydrated in ethanol, cleared in xylene, and mounted using standard mounting medium. Histological evaluation was performed using an Olympus BX53 upright digital microscope equipped with an Olympus DP28 color camera and standard objectives (10×, 20×, and 40×). Different magnifications were used for routine diagnostic assessment and histopathological interpretation. However, all images used for digital documentation and quantitative analysis were acquired at 20× magnification to ensure consistency with the GTEx reference dataset and allow direct morphological comparison.

To facilitate computational analysis, WSIs were processed using the OpenSlide library (Goode et al. 2013). The original SVS‐format WSIs were converted into DeepZoom image pyramids to enable efficient image tiling (https://github.com/openzoom/deepzoom.py). Non‐informative regions were excluded, and image patches were extracted and saved in JPEG format for further processing. To ensure consistency across samples, H&E‐stained images were normalized using the Macenko method (Macenko et al. 2009), which applies color deconvolution to separate hematoxylin and eosin channels, followed by intensity standardization to minimize staining variability across slides. During preprocessing, images containing excessive white space were removed to ensure that the final dataset included only histologically informative regions suitable for downstream analysis.

### 2.2 DAM‐CNN

A residual neural network (ResNet34), previously demonstrated to provide superior performance compared to other network architectures on similar image‐based classification tasks, was adopted as the backbone of our proposed DAM‐CNN architecture (He et al. 2016). Each residual block within the ResNet backbone was enhanced by incorporating a Dual Attention Module DAM.

The dual attention mechanism comprises two complementary components: channel attention and spatial attention (Woo et al. 2018). The channel attention module enhances informative feature channels by modeling inter‐channel dependencies, while the spatial attention module focuses on salient spatial regions within the feature maps. Together, this dual attention mechanism enables the model to capture discriminative information across both the channel and spatial dimensions. A schematic overview of the DAM architecture is presented separately.

For the channel attention module, given an intermediate feature map $F\in {\mathbb{R}}^{C\times H\times W}$ as input, global average pooling and global max pooling operations are applied separately, generating two distinct channel descriptors: $F_{\mathrm{avg}}^{c}$ and $F_{\max}^{c}$:

(1) $$F_{\mathrm{avg}}^{c}=\text{AvgPool}\left(F\right),\;F_{\max}^{c}=\text{MaxPool}\left(F\right)$$

Both descriptors are subsequently processed by a shared multilayer perceptron (MLP). The outputs are combined using element‐wise summation, followed by sigmoid activation, producing the channel attention weights $F_{\text{out}}^{c}$:

(2) $$F_{\text{out}}^{c}=\sigma \left(\mathrm{MLP}\left(F_{\mathrm{avg}}^{c}\right)+\mathrm{MLP}\left(F_{\max}^{c}\right)\right)$$

The recalibrated feature map $F^{\prime }$ is then obtained through element‐wise multiplication of the original feature map F and the channel attention weights:

(3) $$F^{\prime }=F⨂{\;F}_{\text{out}}^{c}$$

Next, $F^{\prime }$ is passed into the spatial attention module, where channel‐wise average and max pooling operations are performed to generate two spatial descriptors, $F_{\text{mean}}^{s}$ and $F_{\max}^{s}$:

(4) $$F_{\text{mean}}^{s}={\text{Mean}}_{\text{channel}}\left(F^{\prime }\right),\;F_{\max}^{s}={\mathrm{Max}}_{\text{channel}}\left(F^{\prime }\right)$$

These two spatial descriptors are concatenated and processed by a two‐dimensional convolutional layer, followed by sigmoid activation, resulting in the spatial attention map $F_{\text{out}}^{s}$:

(5) $$F_{\text{out}}^{s}=\sigma \left(\text{Conv}2D\left(\left[F_{\text{mean}}^{s};F_{\max}^{s}\right]\right)\right)$$

Finally, the output feature map $F^{\prime \prime }$ of the dual attention module is computed via element‐wise multiplication:

(6) $$F^{\prime \prime }=F^{\prime }⨂F_{\text{out}}^{s}$$

Explainable AI and visualization: We utilized Gradient‐weighted Class Activation Mapping (Grad‐CAM, MathWorks, Natick, MA, USA) as an explainable AI method to visualize and interpret model predictions (Gildenblat 2024), particularly attention scores highlighting critical image regions. Feature activations from the final convolutional layer of ResNet34 with the integrated DAM were extracted and visualized using Grad‐CAM to ensure the interpretability and reliability of predictions.

Training Implementation and Computational Resources: The DAM‐CNN model was implemented using the PyTorch deep learning framework (A Paszke 2019; Adam Paszke et al. 2017). Model training was conducted using the Adaptive Moment Estimation (Adam) optimizer over 100 epochs with a learning rate of 1e−5, weight decay of 1e−4, and batch size of 8. All training procedures were performed on a computing workstation equipped with an Intel Xeon Gold 6258R CPU @ 2.70 GHz, 256 GB RAM, and four NVIDIA GeForce RTX 3090 GPUs (NVIDIA Corporation, Santa Clara, CA, USA). Model training required approximately 20 h for completion.

### 2.3 Quantification

For nuclear instance segmentation and quantification, we utilized Detectron2, a state‐of‐the‐art object detection framework developed by Facebook AI Research (FAIR) (https://github.com/facebookresearch/detectron2). Built on top of PyTorch, Detectron2 offers a highly modular and extensible architecture, enabling efficient training and evaluation of various tasks, including object detection, instance segmentation, and keypoint detection (Dang 2023; Paszke 2019).

We fine‐tuned a pre‐trained Mask R‐CNN (Region‐based Convolutional Neural Network) model using the NuInsSeg dataset, which contains fully annotated H&E‐stained histological images designed for nuclear instance segmentation (Mahbod et al. 2024). The dataset comprises a diverse array of tissue types and nuclear morphologies, providing a robust foundation for training a generalized segmentation model. Prior to training, annotations were converted into the COCO JSON format to ensure compatibility with the Detectron2 framework (Dang 2023). Because the analysis was performed on H&E‐stained whole muscle sections without cell‐type‐specific markers, the quantified nuclei represent a mixed population, including myonuclei and non‐myogenic nuclei (e.g., fibroblasts, endothelial cells, and immune cells).

The model architecture employed a ResNet‐50 backbone coupled with a Feature Pyramid Network (FPN) to enhance multi‐scale feature extraction, thereby improving segmentation performance across nuclei of varying sizes (Lin et al. 2017). The training process was conducted for 10,000 iterations. To optimize computational efficiency and model convergence, aspect ratio grouping was applied to cluster training images with similar dimensions during batch construction.

### 2.4 Transcriptomics Analysis

We performed transcriptomic analyses using skeletal muscle RNA‐sequencing data (raw count matrices) obtained from the GTEx consortium. Samples were stratified into two groups according to average nuclear diameter, as measured from our histological analysis. The top and bottom 2.5% of donors—corresponding to those with the largest and smallest nuclei, respectively—were selected, resulting in a final subset of 20 samples for downstream comparisons.

To elucidate molecular pathways associated with nuclear size variation, we first performed differential gene expression analysis, followed by gene set enrichment analysis (GSEA) as previously described (Kim et al. 2022; Subramanian et al. 2005). To further investigate coordinated regulatory activity, enrichment network analysis was conducted using genes identified in the enrichment plots and heatmaps, enabling the visualization of correlated transcriptional patterns between nuclear size groups.

All statistical and visualization procedures were carried out using R (version 4.1.1) within the RStudio environment (version 1.4.1717). The analysis workflow employed several R packages, including *edgeR* for expression analysis, *clusterProfiler* for functional enrichment, and *ggplot2*, *dplyr*, *ggarrange*, and *pROC* for data handling and figure generation. Distribution normality was assessed using the Shapiro–Wilk test. Group comparisons were performed using the Wilcoxon rank‐sum test, Kruskal–Wallis test, or unpaired *t*‐test, depending on data distribution characteristics. Statistical significance was defined as *p*‐values (or adjusted *p*‐values) less than 0.05.

## 3 Results

### 3.1 An Integrated Framework for Discovering Aging‐Associated Histological Features in Skeletal Muscle

**FIGURE 1:**
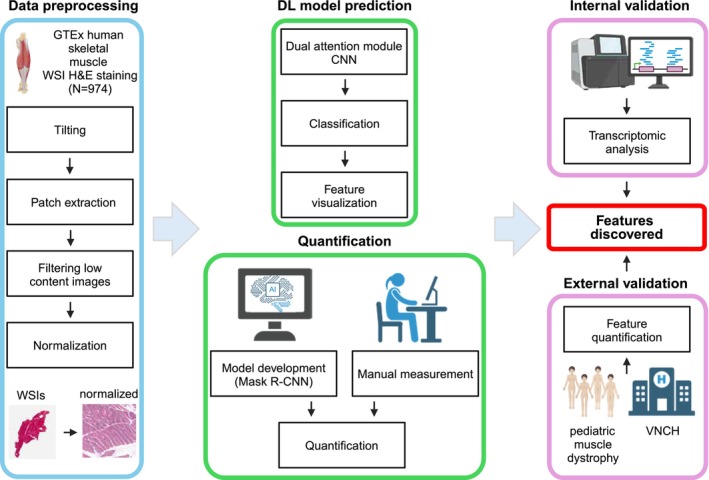
Schematic representation of a deep learning‐integrated framework for identifying age‐associated morphological features in human skeletal muscle. A total of 974 H&E‐stained whole slide images (WSIs) from the GTEx project were curated, corrected for orientation, segmented into high‐content patches, and normalized to ensure consistency for downstream processing. The pipeline includes two parallel components: (1) a classification pathway employing a dual attention module convolutional neural network (DAM‐CNN) to detect and visualize histological patterns associated with aging, and (2) a quantification pathway utilizing a Mask R‐CNN model trained with NuInsSeq‐derived annotations to segment nuclei and extract quantitative features such as size and density, with results cross‐validated through manual measurement. Identified morphological traits were biologically validated via transcriptomic profiling of matched GTEx samples, establishing links between nuclear alterations and gene expression. To confirm clinical relevance, nuclear features were further quantified in pediatric muscle biopsies from patients with muscular dystrophy at the Vietnam National Children's Hospital (VNCH).

To uncover aging‐related morphological features in skeletal muscle, we established a deep learning‐based analytical pipeline combining large‐scale histological image processing with transcriptomic validation (Figure 1).

We first collected 974 H&E‐stained WSIs of human skeletal muscle from the GTEx project (GTEx Consortium 2013), along with donor age metadata. The WSIs were subjected to tilt correction, extraction of high‐content image patches, filtering of low‐information regions, and normalization, thereby ensuring standardized inputs for computational analysis.

Following image preprocessing, the analytical workflow proceeded through two parallel yet complementary pathways. In the deep learning prediction branch, a DAM‐CNN was implemented to classify muscle morphology and visualize histological features associated with aging. In the quantification branch, a Mask R‐CNN was trained using NuInsSeq‐derived annotations to segment and quantify nuclear features such as size and density. Manual measurements were also performed to validate segmentation results and enhance the reliability of nuclear quantification.

To assess the biological relevance of the identified features, matched GTEx samples underwent transcriptomic profiling, which revealed significant correlations between morphological alterations and gene expression patterns. This integrative analysis confirmed the molecular significance of image‐derived histological traits.

External validation was then conducted using skeletal muscle biopsy samples from pediatric patients with muscular dystrophy at the Vietnam National Children's Hospital to evaluate generalizability and clinical applicability. In these samples, consistent nuclear enlargement was observed, reinforcing its role as a reproducible histological marker of both pathological and aging‐associated muscle atrophy.

This integrated framework, combining deep learning‐based morphological classification, quantitative nuclear analysis, and transcriptomic validation, offers a robust and scalable approach for identifying tissue‐level biomarkers of aging and provides a foundation for future diagnostic and therapeutic strategies in muscle degenerative diseases.

### 3.2 Identification of Age‐Associated Nuclear Alterations in Skeletal Muscle by a Deep Learning Model, DAM‐CNN

**FIGURE 2:**
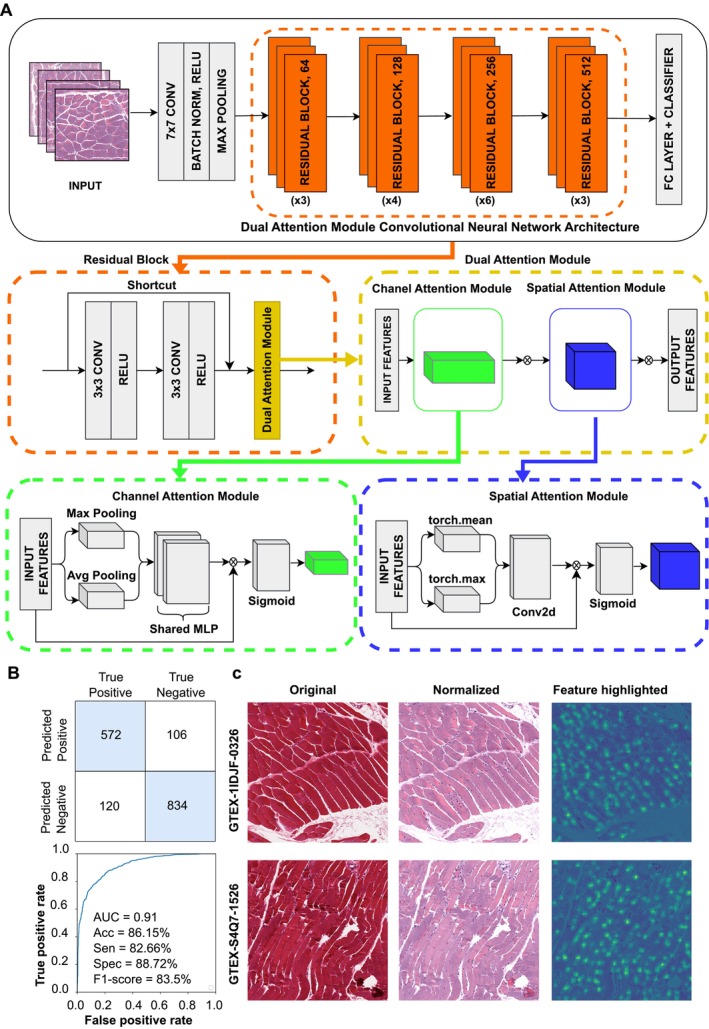
Architecture and performance of the dual attention module convolutional neural network (DAM‐CNN) for age‐associated histological feature prediction (A) Schematic overview of the DAM‐CNN model architecture. The input image patches undergo convolution, batch normalization, ReLU activation, and max pooling, followed by a series of residual blocks with increasing filter depths. A dual attention module, comprising both channel and spatial attention mechanisms, is integrated within each residual block to enhance feature representation. The final output is passed through a fully connected layer for classification. (B) Confusion matrix and receiver operating characteristic (ROC) analysis demonstrate model performance on the test set, achieving an area under the curve (AUC) of 0.91, accuracy of 86.15%, sensitivity of 82.66%, specificity of 88.72%, and *F*1‐score of 83.5%. (C) Representative examples of histological image patches before and after normalization, with corresponding attention maps highlighting regions identified as age‐related features by the trained model.

To identify histological features predictive of muscle aging, we implemented a DAM‐CNN and applied it to standardized skeletal muscle image patches extracted from GTEx WSIs (Figure 2A). The proposed network architecture begins with an initial convolutional layer followed by max pooling and progresses through ResNet34 residual blocks. Each residual block integrates a dual attention module comprising channel and spatial attention mechanisms. The channel attention module captures inter‐channel relationships by applying global average and max pooling across spatial dimensions, followed by a shared multi‐layer perceptron and sigmoid activation to adaptively recalibrate channel‐wise feature responses. In parallel, the spatial attention module models spatial dependencies by applying average and max pooling along the channel axis, concatenating the resulting descriptors, and processing them through a 2D convolution and sigmoid activation to generate a spatial attention map that highlights informative regions (Figure 2A). This architecture enabled the model to simultaneously focus on both spatial and channel information, enhancing its ability to detect subtle age‐associated histological patterns.

For training and evaluation, the dataset was randomly partitioned into an 80% training‐validation set and a 20% test set. Five‐fold cross‐validation was applied to the training‐validation set to ensure model robustness. A binary classification task was conducted to distinguish young from old muscle samples based solely on histological features. Model performance was evaluated using key metrics including accuracy, sensitivity, specificity, and area under the receiver operating characteristic curve (AUC). The resulting confusion matrix indicated a sensitivity of 82.66% ± 1.53%, specificity of 88.72% ± 1.47%, and overall classification accuracy of 86.15% ± 4.85%, with an AUC of 0.91, demonstrating robust discriminatory performance (Figure 2B).

To visualize morphological features contributing significantly to the model's predictions, attention‐based feature maps were extracted from the final convolutional layers. Representative examples include the original H&E image, the normalized input, and the DAM‐CNN‐derived attention map highlighting regions considered most informative by the model (Figure 2C). Notably, highlighted regions frequently corresponded with areas exhibiting nuclear enlargement or irregular nuclear distribution, supporting their relevance to aging‐associated changes in skeletal muscle architecture.

These results demonstrate that the DAM‐CNN model effectively distinguishes between young and aged skeletal muscle based solely on histological patterns, providing spatially interpretable outputs that identify candidate morphological markers such as nuclear size and distribution. Thus, this deep learning framework offers both predictive utility and novel biological insights into histological features underlying skeletal muscle aging.

### 3.3 Quantitative Assessment of Age‐Associated Nuclear Alterations Using Deep Learning‐Based Morphometry

**FIGURE 3:**
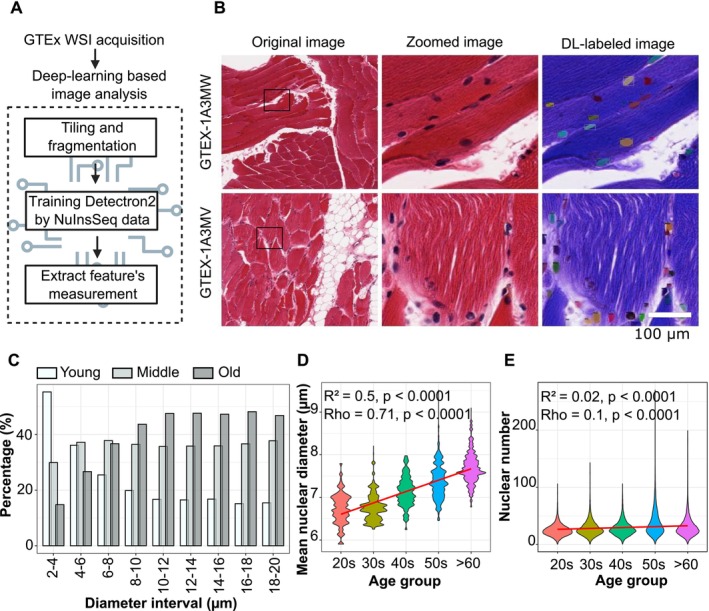
Deep learning‐based quantification of nuclear morphological features in GTEx skeletal muscle samples (A) Workflow illustrating the image analysis pipeline. WSIs were tiled and fragmented, and nuclear segmentation was performed using a model trained with the NuInSeq dataset via the Detectron2 framework to extract morphological measurements. (B) Representative examples from two GTEx samples showing the original H&E‐stained images, zoomed‐in regions, and deep learning‐labeled outputs highlighting detected nuclei. (C) Histogram of nuclear diameter distributions categorized by age group, showing a shift toward larger nuclear sizes in older individuals. (D) Violin plot of mean nuclear diameter across age groups, showing an increase in nuclear size with advancing age. (E) Violin plot of nuclear counts per patch across age groups, showing relatively increased nuclear numbers throughout aging.

To further validate and quantify the histological alterations identified by the DAM‐CNN model, we constructed a deep learning‐based morphometric analysis pipeline designed for high‐throughput measurement of nuclear features in skeletal muscle tissue. As illustrated in the schematic diagram, the pipeline began with WSI acquisition from the GTEx skeletal muscle dataset, followed by image tiling and fragmentation to facilitate computational tractability. A Detectron2‐based instance segmentation model was trained using annotations from the NuInsSeq dataset, which enabled precise detection and segmentation of individual nuclei. The pipeline then extracted key morphological features—including nuclear diameter and number—for quantitative analysis. This automated framework offered scalability across hundreds of samples while minimizing observer bias and standardizing feature extraction across diverse tissue conditions (Figure 3A).

Representative results from the segmentation model applied to GTEx samples are shown as side‐by‐side comparisons of original, zoomed‐in, and model‐labeled histological images, where individual nuclei were successfully identified and labeled across varying structural contexts (Figure 3B). The model robustly detected nuclei in both compact and fibrotic regions, and qualitative visual inspection confirmed that nuclear identification was not disrupted by background staining or tissue artifacts, supporting the validity of automated quantification.

The extracted nuclear features were analyzed across six age groups: 20s, 30s, 40s, 50s, and over 60 years. The distribution of nuclear diameter revealed clear age‐associated shifts, with younger samples enriched for small‐diameter nuclei (2–8 μm), and older samples showing a rightward skew toward larger nuclei, particularly those in the 12–18 μm range (Figure 3C). This trend supports the notion that progressive nuclear enlargement occurs with aging in skeletal muscle.

To quantify this trend more directly, we calculated the mean nuclear diameter across age groups. A strong positive correlation was observed (*R* ^2^ = 0.5, *p* < 0.0001; Spearman's *ρ* = 0.71), indicating that nuclear enlargement is a continuous and significant histological feature of aging (Figure 3D). These findings suggest that nuclear size may reflect underlying cellular processes such as chromatin remodeling or transcriptional deregulation associated with tissue aging.

We also assessed the total nuclear count per image to determine whether nuclear number varies with age. Although a statistically significant association was observed (*R* ^2^ = 0.02, *ρ* = 0.1, *p* < 0.0001), the correlation was weak, and age‐related differences were modest. Interestingly, a transient increase in nuclear number was detected in the fifth decade, followed by a decline in individuals over 60 years of age (Figure 3E). This biphasic pattern may indicate midlife compensatory myonuclear recruitment in response to early muscle degeneration, with subsequent regenerative failure in advanced age.

Together, these results validate nuclear enlargement as a robust and quantifiable morphological hallmark of muscle aging. The deep learning‐based morphometric pipeline enabled scalable, unbiased, and reproducible evaluation of skeletal muscle histology across a wide age range, offering new opportunities to investigate the structural basis of age‐associated muscle decline.

### 3.4 Validation of Nuclear Morphometry Using Manual Measurement From Artifact‐Free Muscle Regions

To further validate the automated nuclear quantification and mitigate potential artifacts introduced by fatty infiltration or fibrosis in aged skeletal muscle, we performed manual nuclear morphometry in carefully selected regions devoid of histological abnormalities. This complementary approach enabled high‐fidelity measurement of nuclear size and number, independent of automated segmentation assumptions and potential confounding by age‐related tissue degeneration.

**FIGURE 4:**
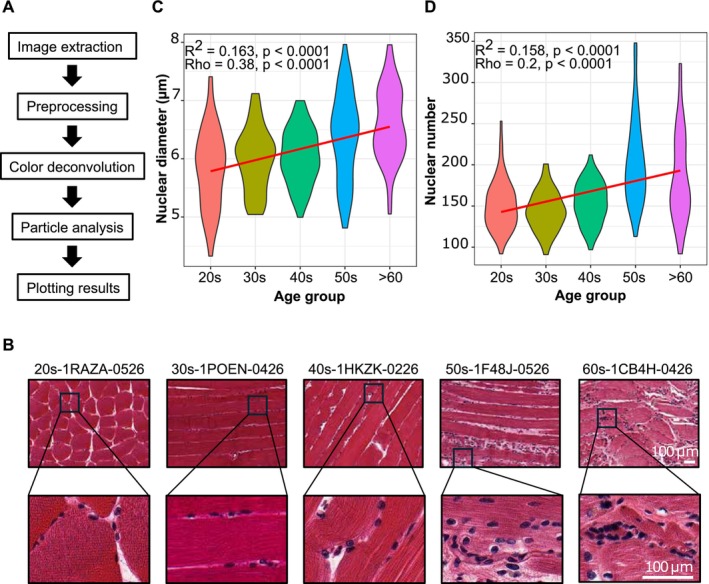
Manual measurement of nuclear features confirms aging‐related morphological and density changes in skeletal muscle (A) Schematic workflow for manual analysis of nuclear morphology. Following image extraction and preprocessing, color deconvolution was applied to isolate hematoxylin staining, and particle analysis was used to quantify nuclear size and number. (B) Representative H&E‐stained muscle sections selected for analysis from individuals across five age groups. (C) Violin plot of nuclear diameter by age group, showing an upward trend in nuclear size with increasing age. (D) Violin plot of nuclear number per field of view across age groups, illustrating an increase in nuclear count with age.

The manual analysis workflow included sequential steps of region selection, image preprocessing, H&E color deconvolution, and particle‐based nuclear detection, followed by statistical summarization of nuclear features (Figure 4A). This pipeline allowed selective analysis of relatively healthy muscle areas across representative samples from different age groups, minimizing bias from pathological structures.

Representative H&E‐stained images illustrate consistent image quality across ages, with magnified insets demonstrating clear nuclear morphology used for measurement (Figure 4B). Importantly, these images confirmed the preservation of nuclear contours and staining quality, enabling accurate size estimation based on nuclear boundaries.

Manual measurement results confirmed age‐associated nuclear enlargement. Violin plots revealed a progressive increase in mean nuclear diameter from the 20s to > 60s age group, consistent with the automated findings. Although the magnitude of correlation was modest (*R* ^2^ = 0.163, Spearman's *ρ* = 0.38), the directionality and statistical significance (*p* < 0.0001) reinforced the robustness of nuclear enlargement as a morphological hallmark of aging (Figure 4C).

Similarly, nuclear number showed a gradual increase across age groups, with older individuals displaying higher nuclear counts per selected region. While the effect size was small (*R* ^2^ = 0.158, *ρ* = 0.20, *p* < 0.0001), the overall pattern paralleled the trend observed in the automated pipeline, supporting its biological plausibility (Figure 4D).

These manually curated findings corroborate the results derived from deep learning‐based segmentation, confirming that nuclear enlargement is a reproducible and quantifiable feature of aging skeletal muscle. Moreover, the consistency across independent pipelines—one automated and comprehensive, the other manual and artifact‐excluding—provides robust validation of nuclear morphometry as a reliable histological indicator of age‐related muscle remodeling.

### 3.5 Transcriptomic Enrichment Analysis Confirms Functional Relevance of Nuclear Size in Muscle Aging

To determine whether nuclear size—identified as a key histological feature by our deep learning model—reflects biologically meaningful aging processes rather than random morphological variation, we performed gene set enrichment analysis (GSEA) using GTEx skeletal muscle transcriptomes (Figure S1A). Specifically, donors were ranked based on the average nuclear diameter quantified from their H&E‐stained muscle WSIs. The top 2.5% (*n* = 20) with the largest nuclei and the bottom 2.5% (*n* = 20) with the smallest nuclei were selected for GSEA comparison (Figure S1B), thereby enabling assessment of transcriptomic signatures associated with morphological extremes in nuclear size.

**FIGURE 5:**
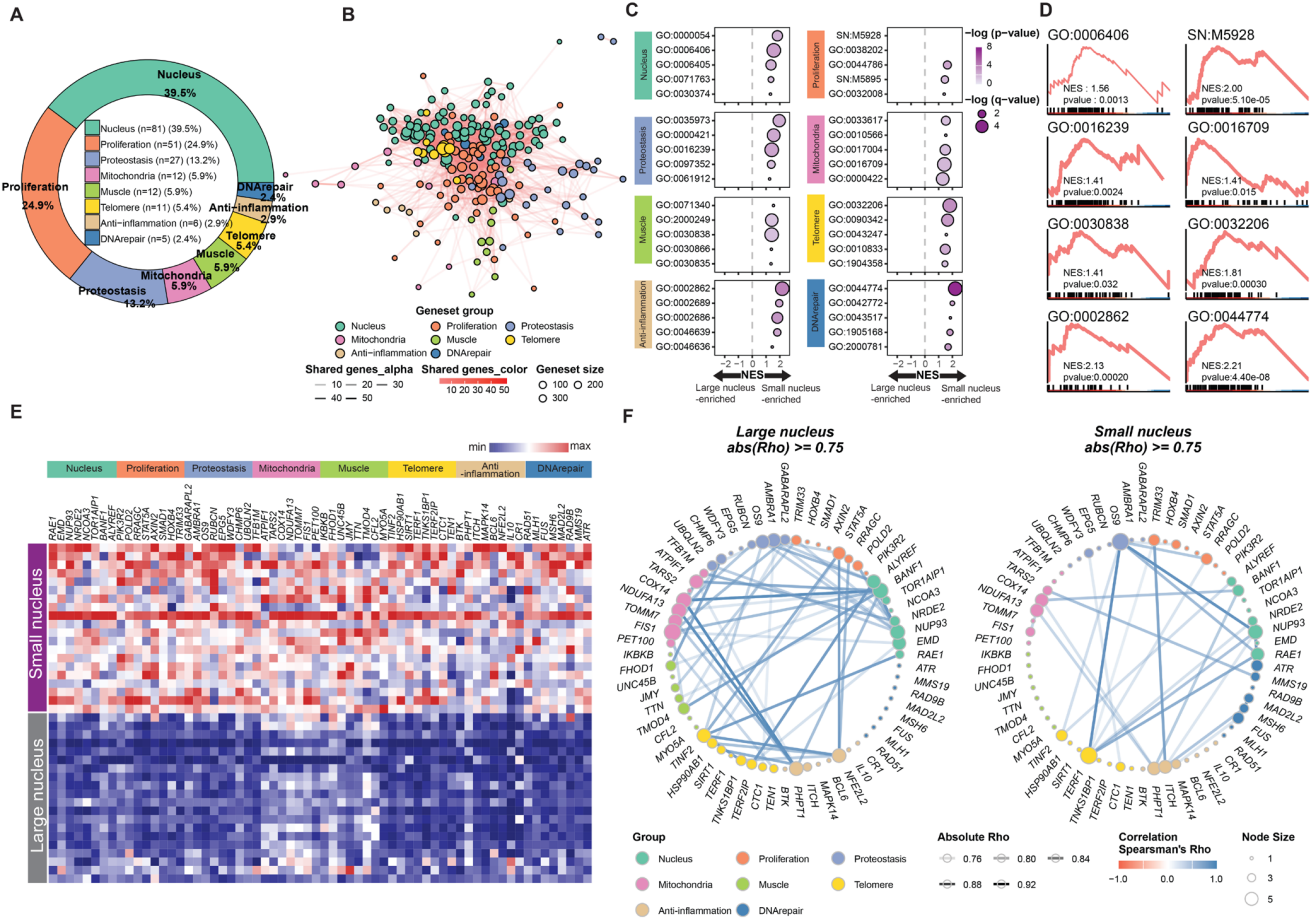
Functional enrichment and correlation analysis of genes associated with nuclear size in human skeletal muscle (A) Donut chart of functional gene categories, highlighting the proportion of genes related to nuclear function, proliferation, proteostasis, mitochondria, muscle, telomere maintenance, anti‐inflammation, and DNA repair. (B) Network representation of gene set interactions. (C) Dot plot of gene set enrichment analysis (GSEA), comparing functional enrichment between large and small nuclei, with normalized enrichment scores (NES) and statistical significance indicated. (D) Representative enrichment plots of key gene sets, showing enrichment scores and *p*‐values. (E) Heatmap displaying the expression of key genes across samples with large and small nuclei, categorized by functional groups. (F) Correlation networks of genes that are highly associated with large and small nuclei (absolute Spearman's rho ≥ 0.75), where node colors indicate functional categories, edge thickness represents correlation strength, and node size corresponds to the degree of connectivity.

To interpret the results in a functional context, enriched gene sets were grouped into eight major biological categories: nuclear function, proliferation, proteostasis, mitochondrial activity, muscle maintenance, telomere regulation, anti‐inflammation, and DNA repair. Among these, nuclear structure and proliferation‐related pathways were the most prominent, accounting for 39.5% and 24.9% of all enriched gene sets, respectively (Figure 5A). This functional distribution underscores the close association between nuclear morphology and cellular programs governing chromatin organization, cell turnover, and protein homeostasis in aging muscle.

To further understand the interrelatedness of enriched gene sets, a network plot was generated based on the degree of gene overlap across categories (Figure 5B). Clusters of co‐enriched gene sets revealed tightly connected modules involving nucleus‐related and proteostasis pathways, as well as senescence‐associated transcriptional programs, suggesting that nuclear enlargement coincides with broader transcriptomic remodeling associated with muscle aging.

Representative gene sets from each functional category demonstrated consistent enrichment patterns between groups. Gene sets across all functional categories—including nuclear envelope, chromatin remodeling, proteostasis, senescence, anti‐inflammatory, and DNA repair pathways—were significantly enriched in samples with small nuclei (Figure 5C). The directionality and magnitude of enrichment were further confirmed using enrichment score plots for selected gene sets, validating the transcriptomic divergence between large and small nucleus muscle samples (Figure 5D).

To determine whether these enriched gene sets were reflected at the gene level, we examined the expression patterns of representative genes within each category. Heatmap analysis revealed that representative genes across all categories—including nuclear architecture, proteostasis, mitochondrial function, DNA repair, and anti‐inflammatory responses—were upregulated in small nucleus samples relative to large nucleus samples (Figure 5E). These findings suggest that muscle with small nuclei maintains a transcriptionally active profile encompassing both homeostatic and stress‐responsive programs, while large‐nucleus muscle exhibits widespread transcriptional downregulation, potentially reflecting aging or senescence‐like states.

Finally, correlation network analysis was conducted to evaluate inter‐gene connectivity within each group. In small nucleus samples, gene–gene correlations were sparse and less organized. In contrast, samples with large nuclei exhibited a tightly interconnected network, particularly among genes associated with nuclear structure, proteostasis, and proliferation (Figure 5F). These results indicate that aging‐associated nuclear enlargement is accompanied by the coordinated activation of molecular pathways implicated in muscle remodeling, cellular senescence, and structural maintenance.

Collectively, these transcriptomic analyses demonstrate that nuclear size, as identified through deep learning‐based image analysis, is not a passive morphological byproduct but a marker closely linked to biologically meaningful gene expression programs. The findings reinforce the interpretation that nuclear enlargement is a feature of aging skeletal muscle with functional implications and further validate the utility of deep learning in discovering histological phenotypes reflective of underlying molecular states.

### 3.6 Nuclear Enlargement as a Premature Aging Marker in Inflammatory Pediatric Myopathies

**FIGURE 6:**
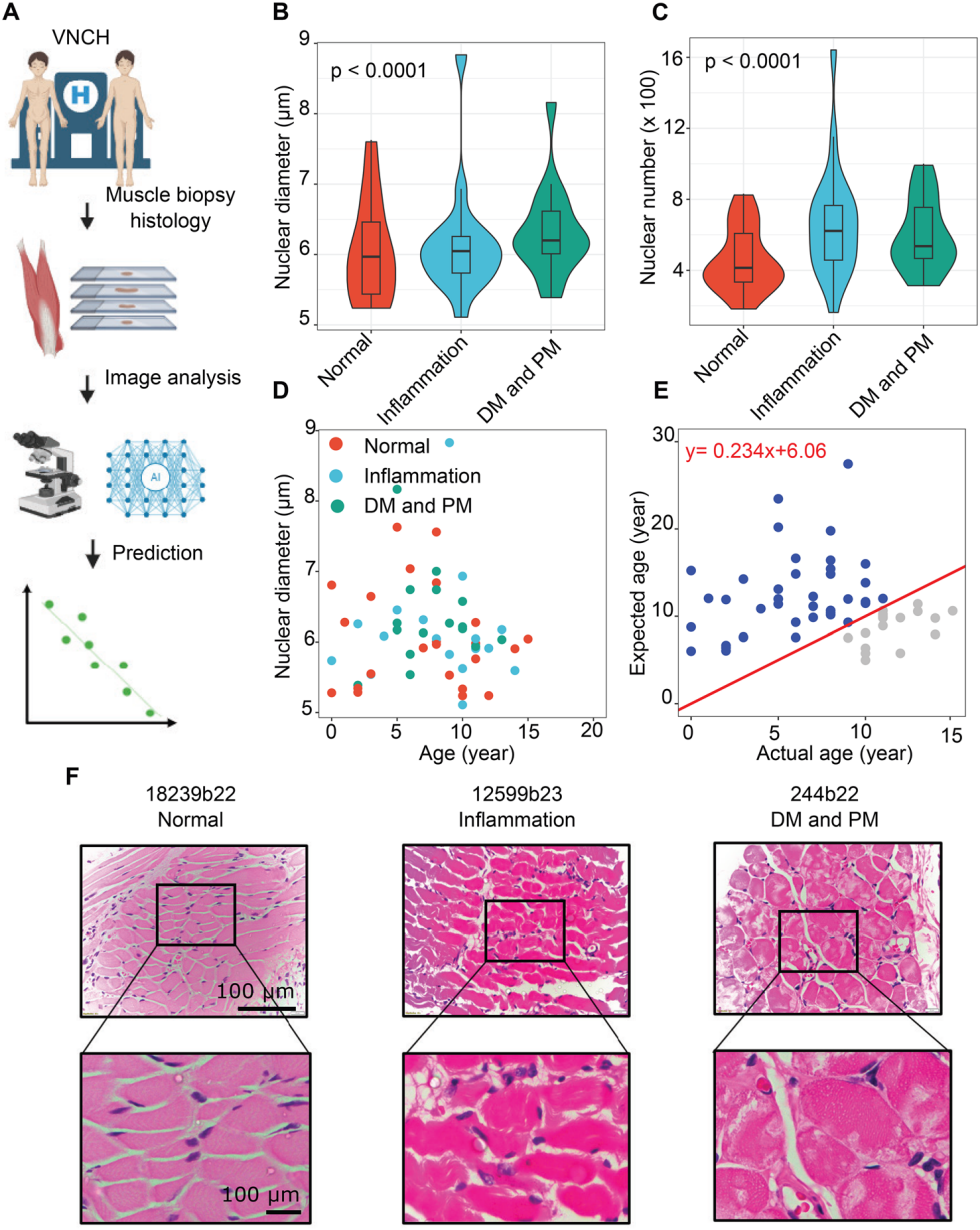
Application of nuclear morphometry to pediatric muscle biopsies reveals premature aging features in inflammatory myopathies (A) Schematic overview of the external validation workflow. Muscle biopsy specimens from pediatric patients at the Vietnam National Children's Hospital (VNCH) were histologically processed, followed by nuclear feature extraction and age prediction using the established deep learning model. (B) Violin plot of nuclear diameter across clinical groups, showing higher values in inflammation and DM/PM groups compared to normal controls (*p* < 0.0001). (C) Nuclear number per image is elevated in both disease groups relative to controls (*p* < 0.0001). (D) Scatter plot of nuclear diameter versus age group, fitted with a linear regression model (*y* = 0.234*x* + 6.06), including data points from normal, inflammation, and DM/PM groups. (E) Predicted biological age plotted against chronological age. Blue points indicate samples with predicted age exceeding chronological age; gray points represent samples with lower or equivalent predicted age. (F) Representative H&E‐stained images from three individuals, one from each group. Lower panels show zoomed‐in regions of the boxed areas in the upper panels. Scale bars: 100 μm.

Skeletal muscle aging is closely associated with chronic inflammation; similarly, juvenile dermatomyositis (JDM) and juvenile polymyositis (JPM) exhibit prominent muscle atrophy and functional decline, together with signs of inflammaging—a chronic, low‐grade inflammatory state increasingly recognized as a hallmark of aging (Gallay et al. 2020; Hohensinner et al. 2011; Jagtap et al. 2022; Lexell et al. 1988). These pathological features partially overlap with histological characteristics of aged muscle, including nuclear enlargement, regenerative remodeling, and altered cellular composition. Based on this conceptual link, we hypothesized that pediatric patients with JDM or JPM might display premature aging phenotypes in skeletal muscles, characterized by enlarged nuclei. To test this hypothesis, we applied our nuclear morphometry pipeline to diagnostic muscle biopsy specimens collected from pediatric patients at the Vietnam National Children's Hospital (VNCH) (Figure 6A).

The biopsy samples were classified into three clinical groups comprising histologically normal tissue, non‐specific inflammatory conditions, and autoimmune myopathies including JDM and JPM. Patients in the histologically normal group initially presented clinical suspicion of neuromuscular disease but were later re‐evaluated and assigned alternative non‐myopathic diagnoses, in accordance with ethical research standards. Morphometric analysis revealed a significant increase in nuclear diameter in both the inflammation and JDM/JPM groups compared to normal controls (*p* < 0.0001), suggesting that nuclear enlargement is a common feature of pathological muscle remodeling (Figure 6B). In addition, the nuclear number per image was significantly elevated in the disease groups (*p* < 0.0001), indicating increased nuclear density, likely resulting from regenerative activity, immune infiltration, or nuclear reorganization (Figure 6C).

We next examined whether nuclear diameter correlated with chronological age in this pediatric cohort. A significant positive association was observed, with nuclear diameter increasing with age, fitting a linear regression model (*y* = 0.234*x* + 6.06) (Figure 6D). Notably, many JDM/JPM samples exhibited nuclear sizes exceeding those of age‐matched controls, reaching values comparable to those observed in adult GTEx donors, supporting the presence of accelerated histological aging in disease states.

To assess whether nuclear morphology could serve as a biological age indicator, we applied our previously developed nuclear feature‐based prediction model to this external dataset. Predicted biological age showed strong concordance with chronological age (Figure 6E). Importantly, samples from the JDM/JPM group tended to cluster above the identity line, indicating that the biological age inferred from nuclear morphology was elevated relative to actual age—a pattern consistent with premature aging at the tissue level.

Representative histological images from each group visually corroborated the quantitative findings. Normal muscle samples exhibited well‐organized fibers with peripheral nuclei, whereas muscle from the JDM/JPM and inflammation groups displayed fiber disorganization, centralized nuclei, and increased nuclear clustering (Figure 6F). These structural alterations are consistent with both inflammatory damage and age‐associated muscle degeneration.

Taken together, these findings validate the robustness and transferability of our deep learning‐based nuclear morphometry framework to independent, clinically relevant tissue samples. Furthermore, they suggest that nuclear enlargement in inflammatory and autoimmune myopathies may reflect a premature aging‐like phenotype, underscoring shared morphological and molecular mechanisms linking disease‐associated and physiological muscle aging.

## 4 Discussion

In this study, we developed a deep learning–based analytical framework to identify histological features associated with aging in human skeletal muscle. Leveraging large‐scale H&E‐stained WSIs from the GTEx project, our DAM‐CNN consistently identified nuclear enlargement as a prominent morphological hallmark of muscle aging. This finding was quantitatively validated through automated nuclear morphometry and manual measurements, and showed a strong relationship with chronological age. Notably, we interpret nuclear enlargement as an aging‐associated feature, rather than a specific or exclusive marker of aging. Notably, we interpret nuclear enlargement as an aging‐associated feature rather than a specific or exclusive marker of aging, reflecting a robust tissue‐level indicator of aging‐related change.

Transcriptomic analyses provided complementary insights, revealing that nuclear enlargement reflects biologically meaningful molecular changes rather than passive structural alterations. Gene sets related to nuclear envelope organization, chromatin remodeling, proteostasis, senescence, anti‐inflammatory responses, and DNA repair were consistently enriched in samples with smaller nuclei. At the gene level, representative pathways across these categories showed higher expression in small‐nucleus samples, suggesting a more transcriptionally active state encompassing both homeostatic and stress‐responsive programs. In contrast, samples with enlarged nuclei exhibited comparatively reduced expression of these pathways, consistent with a decline in coordinated transcriptional activity associated with aging‐ or senescence‐like states. These results indicate that nuclear morphology reflects underlying molecular remodeling in aging muscle tissue. However, we acknowledge that these transcriptomic signatures may reflect a combination of cell‐intrinsic changes and shifts in cellular composition, such as increased fibrosis or immune infiltration. Future studies incorporating immunohistochemistry—for instance, PCM1 to identify myonuclei or Lamin A/C for structural integrity—or spatial transcriptomics will be essential to resolve whether these changes occur predominantly in myofibers, satellite cells, or interstitial populations.

Cellular senescence is a complex and context‐dependent process, with hallmarks that vary across cell types and environments. Anatomically, senescent cells often exhibit increased cell size, nuclear enlargement, and altered nuclear architecture, sometimes accompanied by the formation of phase‐separated compartments. However, most of this evidence derives from in vitro systems. In vivo, particularly in structurally constrained tissues like skeletal muscle, global cell enlargement may be masked by neighboring cells and matrix components. In contrast, nuclear morphology remains readily observable and quantifiable in tissue, as shown in our study. These observations suggest that nuclear features may serve as practical and scalable indicators of cellular senescence and tissue aging.

Notably, muscle biopsies from pediatric patients with JDM and JPM exhibited nuclear enlargement comparable to that of aged adults, indicating premature aging‐like histological changes associated with chronic inflammation. We emphasize that this may reflect overlapping mechanisms between disease‐related remodeling and aging‐like processes, rather than a direct equivalence to physiological aging. This finding implies that inflammatory myopathies may accelerate nuclear remodeling processes typically seen with aging. The inverse relationship observed between nuclear size and muscle atrophy further supports the potential utility of nuclear morphometry as a marker of accelerated aging in pathological contexts.

Despite these findings, the mechanisms driving nuclear enlargement remain to be fully elucidated. Candidate pathways include DNA damage responses, chromatin reorganization, and impaired nucleophagy—a selective autophagic process critical for nuclear quality control. Future studies employing multimodal approaches, such as single‐cell transcriptomics and longitudinal tissue analysis, will be essential to determine whether nuclear enlargement plays a causal role or reflects downstream consequences of muscle aging and degeneration.

## 5 Conclusion

Nuclear enlargement represents a robust and quantifiable histological feature of skeletal muscle aging and is associated with underlying transcriptional and structural alterations linked to inflammation, cellular stress, and tissue degeneration. Using a deep learning–driven nuclear morphometry approach, we demonstrate that nuclear size closely correlates with chronological age and disease progression and captures aging‐related alterations across both physiological aging and pathological muscle conditions. These results suggest nuclear morphometry has the potential to serve as a histological indicator or predictive measure for identifying premature aging‐like phenotypes in both physiological and pathological contexts.

The observation of premature nuclear enlargement in pediatric inflammatory myopathies further supports the possibility of overlapping mechanisms between disease‐associated remodeling and aging‐related processes. Additionally, given prior associations between nuclear enlargement and senescence‐related features, our findings raise the possibility that impaired nuclear clearance mechanisms may contribute to nuclear alterations in aging muscle. These perspectives highlight important directions for future mechanistic studies and therapeutic strategies targeting nuclear remodeling in muscle aging and associated diseases to further elucidate the role of nuclear remodeling in muscle aging and disease.

## Author Contributions

**Tam Dao:** conceptualization, methodology, software, formal analysis, investigation, data curation, visualization, writing – original draft, writing – review and editing. **Thanh T. Nguyen:** methodology, formal analysis, investigation, data curation, writing – review and editing. **Gia Minh Hoang:** investigation, resources, data curation, methodology, visualization, writing – review and editing. **Junhyeon Park:** software, validation. **yunju jo:** software, validation. **Thach Hoang Ngoc:** investigation, resources. **Diep Hong Pho:** investigation, resources. **Dien Tran Minh:** investigation, resources. **Emma Anh Ton:** software, methodology, formal analysis. **Sunjae Lee:** validation, project administration, writing – review and editing. **Hyun Jin Kim:** validation, project administration, writing – review and editing. **Vu Chi Dung:** conceptualization, supervision, writing – review and editing. **Jae Gwan Kim:** conceptualization, methodology, supervision, validation, writing – review and editing. **Dongryeol Ryu:** conceptualization, methodology, supervision, funding acquisition, writing – original draft, writing – review and editing.

## Funding

This work was supported by the Ministry of Health and Welfare (RS‐2024‐00507256), National Research Foundation of Korea (RS‐2021‐NR060106).

## Conflicts of Interest

The authors declare no conflicts of interest.

## Supporting information


**Figure S1:** Sample selection strategy for transcriptomic analysis based on nuclear size. (A) Schematic overview of the analysis pipeline. Nuclear diameter was measured from GTEx skeletal muscle hematoxylin and eosin‐stained skeletal muscle whole‐slide images. Based on these measurements, samples with extreme nuclear sizes were selected for transcriptomic analysis. The corresponding skeletal muscle RNA‐seq data from GTEx donors were subjected to gene set enrichment analysis (GSEA) to identify molecular pathways associated with nuclear enlargement. (B) Violin plot showing the distribution of mean nuclear diameters across all GTEx samples with available transcriptomic data (*n* = 818). Samples in the top 2.5% (*n* = 20) and bottom 2.5% (*n* = 20) of nuclear diameter distribution were selected for GSEA (red dots). Remaining samples not included in the analysis are shown in gray.

## Data Availability

The data that support the findings of this study are available in Genotype‐Tissue Expression (GTEx) at https://www.gtexportal.org/home/. These data were derived from the following resources available in the public domain: GTEx Histology Viewer, https://www.gtexportal.org/home/histologyPage. Representative raw images, processed data, and Supporting Information have been deposited in Figshare (link). All clinical data and histological images are subject to strict institutional governance and privacy protection policies. Access to these materials may be granted only upon formal request and approval by the Vietnam National Children's Hospital (VNCH), in accordance with institutional and ethical regulations. *Code Availability Statement*: All analysis code, pretrained models, and relevant implementation details are publicly available on GitHub (link Github). These materials are freely accessible for academic research and reproducibility purposes. Any additional information or clarification required to reproduce the analyses can be obtained from the corresponding author upon reasonable request.
