# Supplementary material for: Nuclear Enlargement as a Histological Hallmark of Skeletal Muscle Aging, Revealed by Deep Learning‐Driven Analysis and Validated in Inflammatory Myopathies
Source: Aging Cell. 2026 Jun 15;25(6):e70577. doi: 10.1111/acel.70577 (PMC13267430; doi:10.1111/acel.70577)
Supplement: Supplementary file 1 — Figure S1: Sample selection strategy for transcriptomic analysis based on nuclear size. (A) Schematic overview of the analysis pipeline. Nuclear diameter was measured from GTEx skeletal muscle hematoxylin and eosin‐stained skeletal muscle whole‐slide images. Based on these measurements, samples with extreme nuclear sizes were selected for transcriptomic analysis. The corresponding skeletal muscle RNA‐seq data from GTEx donors were subjected to gene set enrichment analysis (GSEA) to identify molecular pathways associated with nuclear enlargement. (B) Violin plot showing the distribution of mean nuclear diameters across all GTEx samples with available transcriptomic data (n = 818). Samples in the top 2.5% (n = 20) and bottom 2.5% (n = 20) of nuclear diameter distribution were selected for GSEA (red dots). Remaining samples not included in the analysis are shown in gray. [file ACEL-25-e70577-s001.docx]

**Supplementary data**


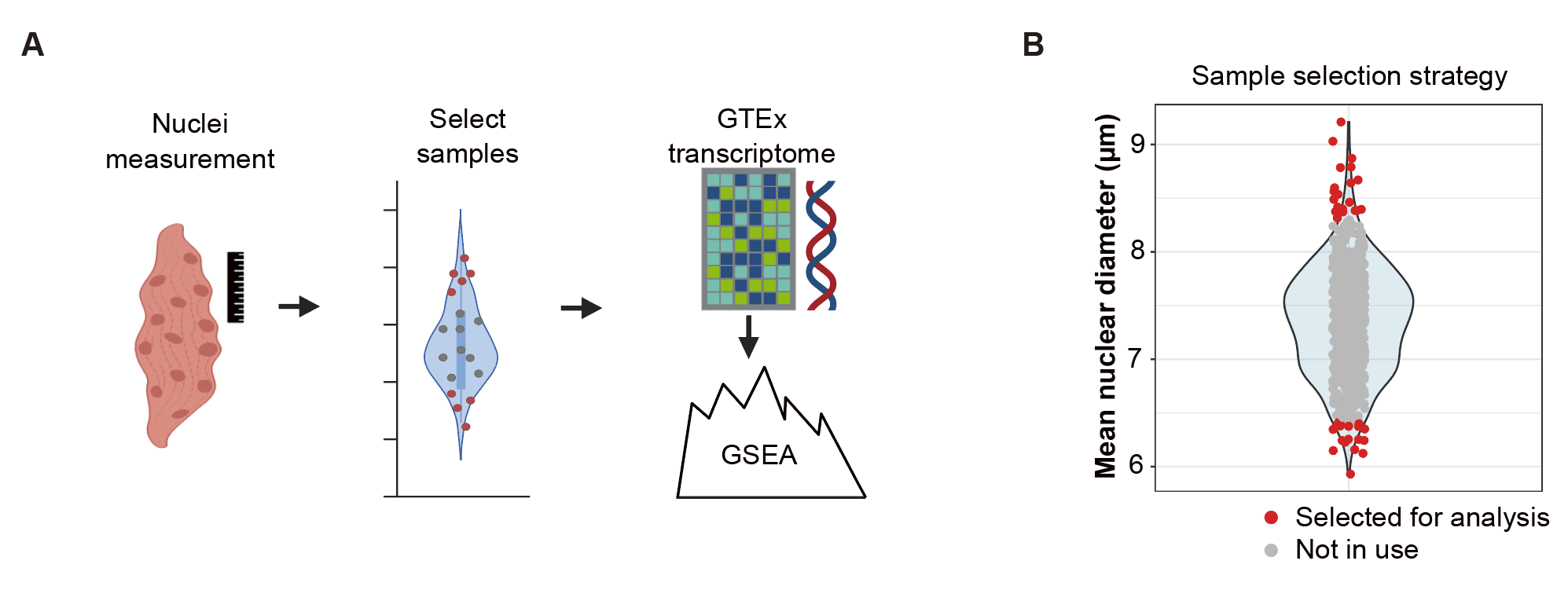


**Figure S1. Sample selection strategy for transcriptomic analysis based on nuclear size.**(A) Schematic overview of the analysis pipeline. Nuclear diameter was measured from GTEx skeletal muscle hematoxylin and eosin-stained skeletal muscle whole-slide images. Based on these measurements, samples with extreme nuclear sizes were selected for transcriptomic analysis. The corresponding skeletal muscle RNA-seq data from GTEx donors were subjected to gene set enrichment analysis (GSEA) to identify molecular pathways associated with nuclear enlargement.
(B) Violin plot showing the distribution of mean nuclear diameters across all GTEx samples with available transcriptomic data (n = 818). Samples in the top 2.5% (n = 20) and bottom 2.5% (n = 20) of nuclear diameter distribution were selected for GSEA (red dots). Remaining samples not included in the analysis are shown in gray.
